# Behavioral and Evolutionary Perspectives on Visual Lateralization in Mating Birds: A Short Systematic Review

**DOI:** 10.3389/fphys.2021.801385

**Published:** 2022-01-31

**Authors:** Masayo Soma

**Affiliations:** Department of Biology, Faculty of Science, Hokkaido University, Sapporo, Japan

**Keywords:** mating, social cognition, visual lateralization, evolution, sexual selection, courtship

## Abstract

The division of cognitive processing between the two hemispheres of the brain causes lateralized eye use in various behavioral contexts. Generally, visual lateralization is shared among vertebrates to a greater extent, with little interspecific variation. However, previous studies on the visual lateralization in mating birds have shown surprising heterogeneity. Therefore, this systematic review paper summarized and analyzed them using phylogenetic comparative methods. The review aimed to elucidate why some species used their left eye and others their right to fixate on individuals of the opposite sex, such as mating partners or prospective mates. It was found that passerine and non-passerine species showed opposite eye use for mating, which could have stemmed from the difference in altricial vs. precocial development. However, due to the limited availability of species data, it was impossible to determine whether the passerine group or altricial development was the primary factor. Additionally, unclear visual lateralization was found when studies looked at lek mating species and males who performed courtship. These findings are discussed from both evolutionary and behavioral perspectives. Possible directions for future research have been suggested.

## Introduction

Neural mechanisms controlling social cognition and sexual interactions in birds cannot be fully understood without knowledge of asymmetries in hemispheric functions. Because avian brain has an asymmetrical structure and lacks major interhemispheric commissure, lateralization of cognitive processing can be seen in every aspect of life of birds (Rogers, 2012), such as sleeping during flight (Rattenborg, 2017), foraging (Alonso, 1998), and social interactions (Vallortigara and Andrew, 1991). This means that looking into lateralized visual behaviors can potentially elucidate both behavioral evolution and its underlying physiological mechanisms.

Lateralized brain function, evident in visual information processing, can have a great impact on how individuals behave in particular situations (Bisazza et al., 1998; Vallortigara et al., 1999; Wiper, 2017; Güntürkün et al., 2020). However, its adaptive significance is still debated (Ghirlanda and Vallortigara, 2004; Rogers et al., 2004; Vallortigara, 2006; Corballis, 2009). Thus far, accumulated evidence has indicated that birds, like many other vertebrates, showed visual lateralization, with biased eye use for particular behavioral tasks or differential behavioral performance based on which eye was used (Vallortigara et al., 1999; Rogers, 2012; Manns and Ströckens, 2014; Wiper, 2017). Specifically, it is well-known that birds generally use the left eye-right hemisphere system for anti-predator search and the right eye-left hemisphere system for foraging, which is consistent with other vertebrates (e.g. Mench and Andrew, 1986; Alonso, 1998; Rogers et al., 2004, 2018). This gives an impression that the pattern of visual lateralization is evolutionarily conserved among avian species.

However, if we focus on the mating context, such as courting, copulation, and pair-bonding interactions between sexes, there is a striking heterogeneity in the previous findings. Some studies reported on right-eye dominance, others on left-eye dominance, or even the lack of clear lateralization (Table 1). For example, males of the black-winged stilt (*Himantopus himantopus*) tend to use their left eye to see a potential mate when performing courtship display (Ventolini et al., 2005), whereas zebra and gouldian finches (*Teniogygia guttata* and *Erythrura gouldiae*) males rely more on their right eye and fail to discriminate appropriate female partners when it is covered with an eyepatch (Templeton et al., 2012, 2014). In contrast, in the greater sage-grouse (*Centrocercus urophasianus*), it is less evident whether the left or right eye is predominantly used by males performing courtship (Krakauer et al., 2016). Such mixed results could partially be due to the differences in experimental designs or specific behavioral contexts. Leliveld et al. (2013) indicated in their review that vertebrates share more or less similar lateralization patterns in emotional processing, including those for inter-sexual interactions, and that right hemisphere generally controls copulation. Based on that, it is suggested that switching from left to right hemisphere would occur in association with the transition from courtship to copulation, as inhibition of the right hemisphere by the left hemisphere takes place during courtship but not copulation (Rogers, 2012; Rogers and Kaplan, 2019). If so, dominant eye is expected to be reversed between courtship and copulation phases. In addition, there could be among-species variations in visual information processing. It should also be noted that previous results might have been overlooked or confounded by sex differences in visual lateralization, if any, as most of them looked at only the male sex (Table 1).

**Table 1:** Summary of the previous findings of visual lateralization in mating birds.

**Species**	**Eye**		**Behavioral context**	**Eyepatch**	**Focal sex**	**Sex effect**	
**Non-passerine**							
White-fronted geese (*Anser albifrons*)	**L**	V	following partner	-	MF	Not tested	^d^
Barnacle geese (*Branta leucopsis*)	**L**	V	following partner	-	MF	Not tested	^d^
Indian peafowl (*Pavo cristatus*)	Unclear^a^	C	wing shaking (courtship display)	-	M	-	^e^
	Unclear^a^	C	train rattling (courtship display)	-	M	-	
Domestic chicken (*Gallus gallus domesticus*)	**R**	V	fixating a model (block)^c^	-	M	-	^f^
	**L**	V	fixating a conspecific (male)^c^	-	M	-	
	**L**	M	copulation (toward human hand)^c^	✓	M	-	^g^
	**L**	M	copulation (toward human hand)^c^	✓	M	-	^h^
Japanese quail (*Coturnix coturnix japonica*)	**L**	V	approaching opposite sex	✓	MF	No	^i^
Wild turkey (*Meleagris gallopavo*)	NS	C	strutting (courtship display)	✓	M	-	^j^
Greater sage-grouse (*Centrocercus urophasianus*)	Unclear^b^	C	strutting (courtship display)	-	M	-	^k^
Black-winged stilt (*Himantopus himantopus*)	**L**	C	bill shaking (courtship display)	-	M	-	^l^
	NS	C	preening (courtship display)	-	M	-	
	NS	C	time spent for courtship	-	M	-	
	**L**	M	mounting	-	M	-	
	**L**	M	time spent for copulation	-	M	-	
**Passerine**							
Gouldian finch (*Erythrura gouldiae*)	**R**	V	viewing potential mates	✓	M	-	^m^
	**R**	C	singing	✓	M	-	
Zebra finch (*Taeniopygia guttata*)	**R**	V	fixating a female	-	M	-	^f^
	NS	V	orienting toward potential mates	-	MF	No	^n^
	**R**	C	singing frequency	✓	M	-	^o^
	**R**	C	singing time	✓	M	-	^p^
Java sparrow (*Lonchura oryzivora*)	**R**	C	courtship dancing	-	MF	No	^q^

a *Inferred based on relative male-female positioning reported*.

b *Left-eye for frontal view and right-eye for lateral view*.

c *Hormone-induced sexual behaviors in young males*.

d *Zaynagutdinova et al. (2021)*.

e *Dakin and Montgomerie (2009)*.

f *Workman and Andrew (1986)*.

g *Rogers et al. (1985)*.

h *Bullock and Rogers (1992)*.

i *Gülbetekin et al. (2007)*.

j *Vernier (2016)*.

k *Krakauer et al. (2016)*.

l *Ventolini et al. (2005)*.

m *Templeton et al. (2012)*.

n *ten Cate et al. (1990)*.

o *George et al. (2006)*.

p *Templeton et al. (2014)*.

q *Endo (2018)*.

*Left (L) or right (R) eye bias is shown with corresponding behavioral context (C, courting, including performing courtship display or singing; M, mounting or copulation; V, viewing, or fixating a potential mate/pairing partner outside the courtship or copulation phase). The use of an eyepatch and the focal sex (M, male; F, female) are also indicated. NS indicates statistically non-significant eye bias*.

To explain the above interspecific variations, a number of ultimate and proximate factors can be applied. First, phylogenetic relatedness itself is a crucial predictor of the presence/absence or direction of visual lateralization in mating contexts (cf. Vallortigara et al., 1999; Brown and Magat, 2011). Given that hemispheric asymmetries are widely conserved among animal species (Güntürkün et al., 2020), it is plausible that evolutionary constraints limit their development to an extent. Second, sexual selection, another ultimate factor, may have an influence. In general, the evolution of sexual signals is inseparable from the sensory systems that process the signals in each species (e.g. Lind and Delhey, 2015; Hiyama et al., 2018; Heffner et al., 2020). If visual lateralization has an advantage in recognizing “good” mates, as shown for foraging tasks (Güntürkün et al., 2000), those species experiencing intense sexual selection (e.g. polygyny) may exhibit strong lateralization, especially in females. Lastly, as a proximate factor, the diversity in the developmental mode, known as precocial-altricial spectrum, can play a role. Due to the asymmetrically turned head position in the egg, the left and right eyes of chicken embryos experience imbalanced light exposure, which is known to determine the ontogeny of lateralized visual behaviors (reviewed in Rogers, 2012; Güntürkün and Ocklenburg, 2017). Similarly, ontogenetic light experience is important in pigeons, although in a different way from that in chickens, which is argued to be caused by differences in altricial (pigeon) and precocial (chicken) neural development (Rogers, 2012; Manns and Ströckens, 2014) (see also Templeton and Gonzalez, 2004).

This review aimed to offer new perspectives by synthesizing past research findings on visual lateralization in mating contexts of a variety of bird species by relying on quantitative interspecific comparative approaches called phylogenetic comparative methods (PCMs) (Garamszegi, 2014; Cornwell and Nakagawa, 2017). Specifically, this approach can allow the estimation of the strength of phylogenetic constraints (i.e., phylogenetic signal) for visual lateralization, the tendency that closely related species resemble each other (Münkemüller et al., 2012). More importantly, the effects of the aforementioned proximate and ultimate factors (mating system and developmental mode) can be statistically evaluated using phylogenetic regression models. Additionally, it was predicted that the methodology (e.g., focal behavioral contexts and the use of an eyepatch) may be partially accountable for the heterogeneity in previous findings.

## Methods

### Literature Data

Data were collected from published research literatures that reported on visual lateralization in the contexts of courting, mate assessment, copulation and pair-formation or -bonding. A search was conducted on Google Scholar using the keyword search bar and terms such as “laterality” “mating” “courtship” and “sexual.” Cross-reference searches were conducted by checking all the cited literature of the papers included. Table 1 presents the data collected from the literature.

**Figure 1:**
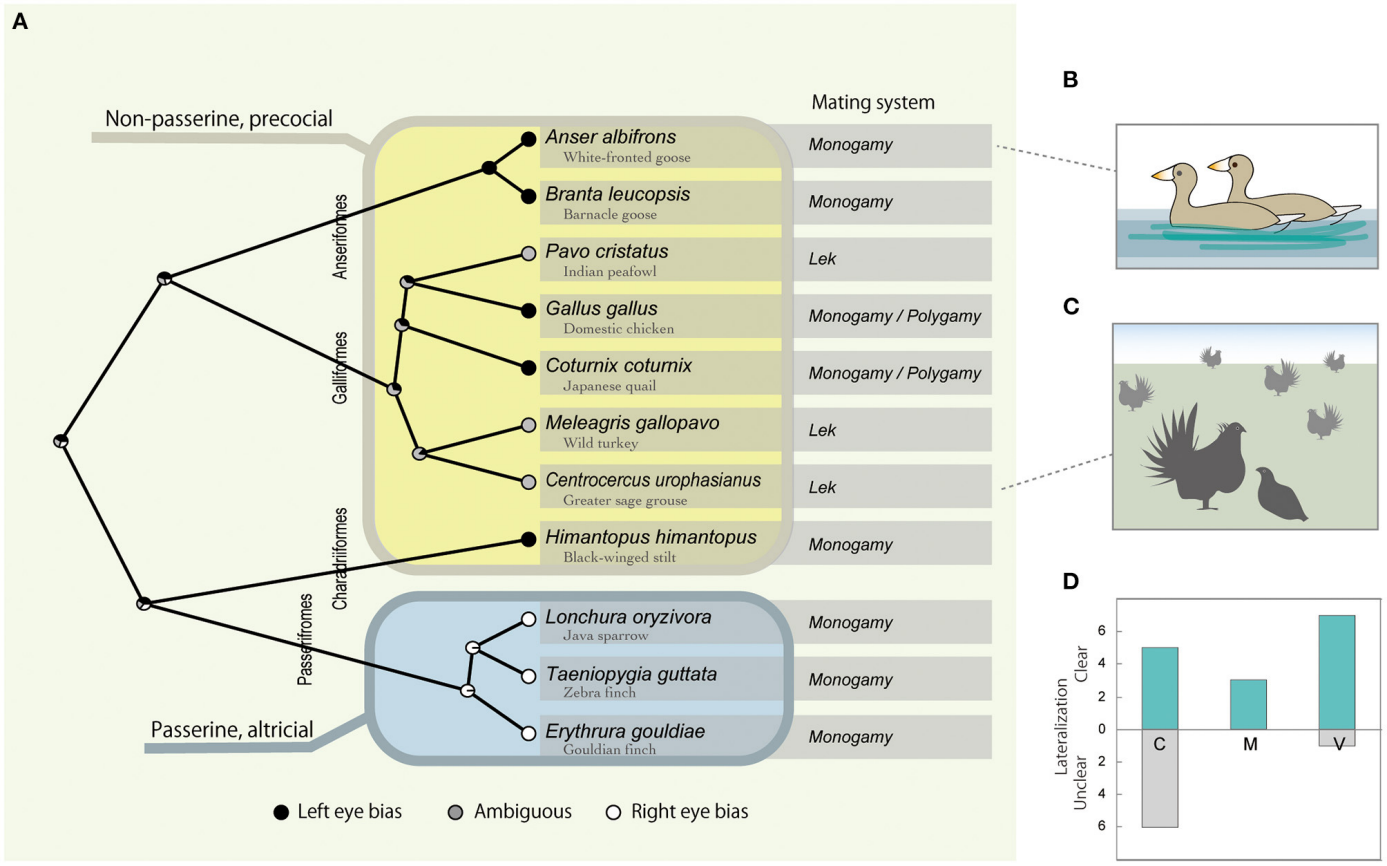
Interspecific variation of the visual lateralization in mating context, with left and right eye bias plotted black and white and ambiguous gray, respectively at each tip of the phylogenetic tree **(A)**. Node pie charts in **(A)** represent the relative probability of left, right, or ambiguous eye bias, inferred by ancestral state reconstruction. Typical examples of the behavioral contexts are given as illustrations, partner viewing in monogamous geese in **(B)**, and courtship display of lekking grouse in **(C)**. Table 1 data are summarized as the number of cases that found clear or unclear lateralization under C (courting), M (mounting) or V (viewing) context in **(D)**.

Based on the results from each study, the presence of visual lateralization and its direction at both the specific behavioral context level (*n* = 23; Table 1) and the species level (*n* = 11; Figure 1A) was determined. Whether one study looked at multiple behavioral contexts, or multiple studies looked at the same species, there was no contradiction regarding the direction of laterality, except for the reports on domestic chickens (Rogers et al., 1985; Workman and Andrew, 1986; Bullock and Rogers, 1992); more repeatable findings that contained species' laterality data were prioritized (Table 1; Figure 1A).

### Phylogeny

For the phylogenetic comparative analyses below, multiple candidate trees were obtained from the Global Phylogeny of Birds database (Jetz et al., 2012). In particular, 1,000 trees were downloaded, the analyses was repeated for each tree, and the model-averaged outcomes were obtained, which have been commonly conducted in recent studies with PCMs (for details see Garamszegi and Mundry, 2014).

### Species-Level Analyses

To assess the evolutionary history of visual lateralization, a maximum-likelihood ancestral state reconstruction was performed and the phylogenetic signal (Pagel's λ) was quantified, which ranged from 0 (no phylogenetic signal) to 1 (strong signal). To examine the effects of factors that covaried with visual lateralization, phylogenetic regression analyses (phylogenetic generalized least-squares, PGLS) was conducted, where the dependent variable was entered as 1: left-eye dominance, −1: right-eye dominance, or 0: unclear. As independent variables, the mating system was scored and entered as 0: monogamy, 1: mixed monogamy, and polygamy, 2: lek breeding, along with the developmental mode binary-categorized as precocial or altricial, based on literature (Goodwin, 1982; Madge and McGowan, 2002) and birds of the world online (https://birdsoftheworld.org/bow/home). The present data did not contain species with semi-precocial or semi-altricial development.

### Behavioral Context-Level Analyses

Under the prediction that the methodological aspects of each study would have an influence on whether clear visual lateralization could be found, another set of phylogenetic regression analyses using the data in Table 1 was performed. To deal with multiple entries per species, generalized linear mixed models (GLMM) were used using the Markov Chain Monte Carlo technique (MCMCglmm, Hadfield, 2010), where the dependent variable was entered as 1: presence or 0: absence of clear visual lateralization in each behavioral context of each species. As explanatory factors, the focal behavioral contexts were considered and categorized as C (courting, which included courtship display or singing, Figure 1C), M (mounting or copulation), or V (just viewing, or fixating a potential mate/pairing partner outside the courtship or copulation phase, Figure 1B), and whether an eyepatch was used to control the available hemifield.

All statistical analyses were conducted using the R ver. 4.1.0, and its package phytools (Revell, 2012), caper (Orme, 2012), phylolm (Ho et al., 2018), and MCMCglmm (Hadfield, 2010).

## Results

### Phylogenetic Signal

Although it should be noted that the previously studied species were limited and belonged to only four avian orders (Anseriformes, Galliformes, Charadriiformes, and Passeriformes), a strong and statistically significant phylogenetic signal for the direction of visual lateralization (average λ = 1.0, *p* < 0.02) with passerine species that showed right-eye bias was found, while the rest showed left -or obscure left/right dominance (Figure 1A). Additionally, the phylogenetic signal was tested for the presence/absence of visual lateralization, but it was relatively weak and not statistically significant (average λ = 0.34, *p* < 0.55).

### Factors Responsible for Visual Lateralization at Species Level

The outcomes from the PGLS indicated that visual lateralization was associated with both the mating system and developmental mode (Supplementary Table S1). Specifically, right-eye bias tended to be observed in monogamous and altricial species and left-eye bias in polygynous and precocial species (Figure 1A). However, this result can be interpreted in different ways. The binary categorization of developmental modes (altricial vs. precocial) also completely matched with passerine vs. non-passerine distinctions, at least for the present species data (Figure 1A). While all passerine birds are altricial, non-passerines show a range of developmental patterns from altricial to precocial. Without the data from altricial non-passerine species (e.g., pigeons, cormorants, and parrots), it was difficult to determine whether being passerine or altriciality was the key factor.

### Factors Responsible for Visual Lateralization at the Behavioral Level

As predicted, focal behavioral contexts had an effect on whether clear visual lateralization was observed (Figure 1D). In particular, compared with V (viewing) and M (mounting and copulation) contexts, clear lateralization was less likely to be observed in the C (courtship) context (Table 1; Supplementary Table S2). The use of an eyepatch did not have a significant influence (Supplementary Table S2).

In addition, Table 1 showed that the left/right eye dominance was not necessarily dependent on particular behavioral context, which was against the prediction that dominant eye would be reversed between courtship and copulation phases (i.e., right eye for courtship and left eye for copulation, Rogers, 2012; Rogers and Kaplan, 2019).

## Discussion

Despite numerous past reviews and empirical studies on avian visual lateralization, to my best knowledge, the present paper was the first attempt to apply phylogenetic comparative methods (PCMs) to systematically integrate previous findings focused on heterogeneous results regarding mating context. Although the outcome interpretations should be done with caution due to the limited availability of species data, the PCMs have revealed overlooked aspects of the evolution of avian visual lateralization. As predicted, the phylogenetic signal for the direction of visual lateralization was quite strong, and passerines (songbirds) and non-passerines (non-songbirds) showed opposite trends in biased eye use, with less variability depending on behavioral contexts. This could potentially be seen as differences in altricial and precocial developments and was also associated with interspecific variations in the mating system. In addition, whether research could find clear visual lateralization was likely to be dependent on the focal behavioral context, as males who performed courtships tended to show less clear laterality, presumably using both eyes.

Reversed visual lateralization between passerine and non-passerine species, as shown in the present study (Figure 1A), may not be as simple as everything being reversed between them, but could be seen as part of complex interspecific variations of visual information processing among birds. For example, previous studies that looked at visual behaviors sensing predator-like stimuli reported that both zebra finches and chicken chicks relied on the left eye-right hemisphere system (Rogers, 2000; Rogers et al., 2018). However, two closely related passerine species showed opposite lateralization within a study (Franklin and Lima, 2001). In contrast, in visual discrimination tasks, passerines and non-passerines were contrasting, as European starling (*Sturnus vulgaris*) relied on the left eye (Templeton and Gonzalez, 2004) while non-passerines, which included not only chicken chicks but also pigeons (*Columba livia*), used the right eye (Mench and Andrew, 1986; von Fersen and Güntürkün, 1990): these are instances where the passerine vs. non-passerine rather than the altricial vs. precocial distinction may apply. Above all, the biggest lesson learnt is that investigating model species, especially chicken chicks alone, may not be sufficient for a synthetic understanding, as Galliformes reflect the features of basal avian species, from which more recently evolved species (e.g., Passeriformes) would have been greatly diverged. Hence, its corresponding mechanisms and evolutionary selective forces are unclear.

It should also be noted that visual behaviors in the mating context are trickier than those in others, such as foraging or detouring, due to the nature of bilateral communication between the sexes. In essence, seeing or being seen is not easily separable during mating. For both males and females, visual information of prospective mates is important, but in different ways. Generally, females carefully assess male quality by relying on morphological or behavioral sexual traits (Andersson, 1994; Byers et al., 2010; Soma and Garamszegi, 2011). Similarly, males may assess females but would rather monitor their responses to adjust their behaviors depending on the feedback from females for better success in copulation or pair formation (e.g. Balsby and Dabelsteen, 2002; Patricelli et al., 2006; Barske et al., 2015). However, this could be reversed in species with sex role reversals (Edward and Chapman, 2011). In such mating interactions, the female's mate choice, or female choosiness usually plays a major role as a driver of sexual selection. This means that most of the past visual lateralization studies summarized in this paper focused on the side that was subject to scrutiny (i.e., males). Indeed, some studies revealed how indiscriminating males could be and showed that testosterone treated chicken chicks normally showed copulatory behaviors, even toward a human hand or a block covered with a yellow towel (Table 1) (Rogers et al., 1985; Workman and Andrew, 1986; Bullock and Rogers, 1992). Due to the scarcity of evidence (Table 1), it is difficult to discuss sex differences in visual behaviors during mating interactions, if any. However, males and females may not differ dramatically in visual lateralization (Güntürkün and Kischkel, 1992; Gülbetekin et al., 2007).

Given the above-mentioned seeing and being seen interaction between the sexes, it makes sense that clear visual lateralization tends to be lacking, particularly for males performing courtship display and not for those viewing or mounting females. In general, courtship displays are highly ritualized and stereotyped within species, which limits the flexibility of the body head orientation and mobility of the males. More importantly, as the nature of sexual signals conveying the physical quality of individuals, courtship behaviors, expressed as vocalizations and/or physical movements, are energetically costly (Vehrencamp et al., 1989; Zollinger et al., 2011; Clark, 2012). Therefore, it might be too challenging to cope with both, showing off the best courtship performance and adjusting positions and postures to trace a female moving around trying to assess the quality of the potential mates (Krakauer et al., 2016).

In addition, seeing and being seen interactions could be more complicated in the social environment of lekking species, which might explain the rather unexpected heterogeneity associated with mating systems found in previous research. As shown in Figure 1, the three previously studied lekking species did not show clear visual lateralization. For example, males of the greater sage-grouse showed left-eye bias when they used the frontal field but not they used the lateral field (Krakauer et al., 2016), which suggested the possibility that they might be monitoring individuals other than the courtship target using the frontal view at times. In lek, by definition, many males gather and are visited by multiple females, where both male-male competition and female attraction affect the mating success of individuals (Figure 1C) (Andersson, 1993; Loyau et al., 2005). Unlike monogamous mating species, where one male—one female interaction is common, lekking males pay attention to rival males and potential courtship targets that are visiting other males. For this, they could rely on the left eye-right hemisphere system, as shown for visual lateralization concerning social information processing in Galliformes and other animals (Deng and Rogers, 2002; Daisley et al., 2009; Salva et al., 2012).

For future directions of research in this area, exploring the following three domains is suggested: courtship vs. copulation, females, and pigeons. Although reversed eye-use is predicted between courtship and copulation phases (Rogers, 2012; Rogers and Kaplan, 2019), only a few studies contrasted the two behavioral contexts within the same species, which limits our understanding of cognitive control of sexually motivated behaviors. Moreover, as already mentioned, the evolution of visual lateralization in mating contests is still unclear due to a lack of species data and less focus on females. In particular, clarifying whether the passerine vs. non-passerine or the altricial vs. precocial distinction is responsible for the interspecific variations in lateralization by studying non-passerine altricial species would provide valuable insights. In addition, it should be noted that the passerine songbirds previously studied were all Estrildid finches (family: Estrildidae), implying that the observed phenomena were specific to a taxonomic group. Lastly and most importantly, I would like to emphasize that all the above viewpoints would lead us to deeper insights into physiological mechanisms of sexual/social behaviors and their evolution in birds. This would be critically important when we try to answer how much cognitive mechanisms related with sexual/social interactions are conserved in birds even while they evolved to diversify developmental patterns, mating systems, and the ways to communicate.

## Data Availability Statement

The original contributions presented in the study are included in the article/Supplementary Material, further inquiries can be directed to the corresponding author.

## Author Contributions

MS collected and analyzed the data and wrote the manuscript.

## Funding

This research was financially supported by a Shiseido Female Researcher Science Grant and Grant-in-Aid for Scientific Research (No. 20K06809) awarded to MS.

## Conflict of Interest

The author declares that the research was conducted in the absence of any commercial or financial relationships that could be construed as a potential conflict of interest.

## Publisher's Note

All claims expressed in this article are solely those of the authors and do not necessarily represent those of their affiliated organizations, or those of the publisher, the editors and the reviewers. Any product that may be evaluated in this article, or claim that may be made by its manufacturer, is not guaranteed or endorsed by the publisher.
