# Supplementary material for: Behavioral and Evolutionary Perspectives on Visual Lateralization in Mating Birds: A Short Systematic Review
Source: Front Physiol. 2022 Jan 31;12:801385. doi: 10.3389/fphys.2021.801385 (PMC8841733; doi:10.3389/fphys.2021.801385)
Supplement: Supplementary file 1 [file Table_1.DOCX]

**Supplementary Materials**

**Table S1.** Effects of the mating system and altricial vs. precocial or passerine vs. non-passerine distinction on the direction of visual lateralization. Each coefficient is estimated from a model averaging over phylogenetic logistic regressions conducted for 1000 phylogenetic trees. Positive coefficient values indicate bias toward left-eye and negative values for right-eye. Bold typeface was used when 95% CI did not include zero, and thus can be interpreted as a significant effect.

|  | Coefficient | 95% CI | | |
| --- | --- | --- | --- | --- |
| Intercept | -0.96 | [-1.49 |  | 0.42] |
| Mating system (0: monogamy, 1: mixed monogamy and polygamy, 2: lek) | **-0.82** | **[-1.03** |  | **-0.62]** |
| Altricial vs. Precocial development  Passerine vs. Non-passerine | **2.21** | **[1.60** |  | **2.82]** |

**Table S2.** Effects of the behavioral contexts and the use of an eyepatch on the presence of clear visual lateralization. Each coefficient was estimated from a model averaging over MCMCglmm for 1000 phylogenetic trees. Bold typeface was used when 95% CI did not include 0, and thus can be interpreted as a significant effect. Significant positive effects are found for mounting and viewing contexts when comparing against the courtship context.

|  | Coefficient | 95% CI | | |
| --- | --- | --- | --- | --- |
| Intercept | -85.6 | [-328.4 |  | 112.5] |
| Context - mounting | **343.3** | **[10.8** |  | **697.2]** |
| Contest - viewing | **242.7** | **[0.35** |  | **564.9]** |
| Use of an eyepatch | 178.1 | [-55.7 |  | 463.0] |
